# Regular Cocaine Use Is Associated with Increased Systolic Blood Pressure, Aortic Stiffness and Left Ventricular Mass in Young Otherwise Healthy Individuals

**DOI:** 10.1371/journal.pone.0089710

**Published:** 2014-04-09

**Authors:** Rebecca Kozor, Stuart M. Grieve, Stefan Buchholz, Sharlene Kaye, Shane Darke, Ravinay Bhindi, Gemma A. Figtree

**Affiliations:** 1 North Shore Heart Research Group, Kolling Institute of Medical Research, University of Sydney, Sydney, Australia; 2 Department of Cardiology, Royal North Shore Hospital, Sydney, Australia; 3 Sydney Translational Imaging Laboratory, Sydney Medical School, University of Sydney, Sydney, Australia; 4 National Drug and Alcohol Research Centre, University of New South Wales, Sydney, Australia; The University of Manchester, United Kingdom

## Abstract

**Background:**

The cardiovascular impact of cocaine use in otherwise healthy individuals who consider themselves ‘social’ users is not well established.

**Methods/Results:**

Twenty regular cocaine users and 20 control subjects were recruited by word-of-mouth. Cardiovascular magnetic resonance was performed to assess cardiac and vascular structure and function. Cocaine users had higher systolic blood pressure compared to non-users (134±11 vs 126±11 mmHg, p = 0.036), a finding independent of age, body surface area, smoking and alcohol consumption. Cocaine use was associated with increased arterial stiffness - reflected by reduced aortic compliance (1.3±0.2 vs 1.7±0.5 cm^2^×10^−2^.mmHg^−1^, p = 0.004), decreased distensibility (3.8±0.9 vs 5.1±1.4 mmHg^−1^.10^−3^, p = 0.001), increased stiffness index (2.6±0.6 vs 2.1±0.6, p = 0.005), and higher pulse wave velocity (5.1±0.6 vs 4.4±0.6 m.s^−1^, p = 0.001). This change in aortic stiffness was independent of vessel wall thickness. Left ventricular mass was 18% higher in cocaine users (124±25 vs 105±16 g, p = 0.01), a finding that was independent of body surface area, and left atrial diameter was larger in the user group than controls (3.8±0.6 vs 3.5±0.3 cm, p = 0.04). The increased left ventricular mass, systolic blood pressure and vascular stiffness measures were all associated with duration and/or frequency of cocaine use. No late gadolinium enhancement or segmental wall motion abnormalities were seen in any of the subjects.

**Conclusions:**

Compared with the non-user control cohort, cocaine users had increased aortic stiffness and systolic blood pressure, associated with greater left ventricular mass. These measures are all well known risk factors for premature cardiovascular events, highlighting the dangers of cocaine use, even in a ‘social’ setting, and have important public health implications.

## Introduction

Cocaine is an illicit drug that is frequently used across a wide range of demographic groups. The acute cardiovascular effects of cocaine are well known and include, but are not limited to: accelerated hypertension [1], acute myocardial ischaemia and infarction [2], aortic dissection [3], and life-threatening arrhythmias [4]. These acute effects are attributed to a number of mechanisms including cocaine's powerful sympathomimetic properties [5], coronary artery vasoconstriction by direct vessel smooth muscle activation [6], the inhibition of endogenous nitric oxide production and the release of potent vasoconstrictors such as endothelin-1 [7]. Cocaine also has an acute effect on arterial thrombosis through a number of mechanisms acting on platelets [8], [9].

The chronic effects of cocaine use are difficult to study, and data is mostly derived from small cohorts of cocaine ‘addicts’ or ‘abusers’ recruited from drug rehabilitation centres that may not represent the average user. In the ‘addict’ population, cocaine use has been associated with myocardial fibrosis [10], [11], impairment in both systolic and diastolic function [12] and left ventricular hypertrophy [13], [14]. However, the chronic effect of regular cocaine use in otherwise healthy adults who consider themselves as ‘social’ users is more difficult to establish. According to the Australian 2010 National Drug Strategy Household Survey report [15], 7.8% of Australians aged 18+ had used cocaine in their lifetime, and 2.1% in the last 12 months. Also, users were predominantly male, aged 20–39 years, currently employed with post-school qualifications, living in major cities, and of the highest socioeconomic status [15]. In Australia, studies examining cocaine users across the Sydney metropolitan area have identified two distinct groups of users – a group with a higher socio-economic status (SES) and a group with a lower SES [16]–[18]. The lower SES group had lower levels of education, higher unemployment, were more criminally active, and had higher levels of cocaine use and diagnosis of cocaine dependence. These users preferred injecting as the means of cocaine administration, and more frequently used cocaine in combination with heroin. The higher SES users had regular or above average incomes and reported quite a different usage profile - typically taking cocaine intra-nasally, usually in conjunction with alcohol, on a recreational basis, considering themselves ‘social’ users rather than ‘addicts’.

Cardiovascular Magnetic Resonance Imaging (CMR) is a non-invasive imaging modality with the ability to characterize myocardium, as well as to accurately measure cardiac and vascular function [19]. A specific advantage of CMR that is especially relevant to this study is the ability to detect focal fibrosis and silent myocardial infarction (MI) through the use of late gadolinium enhancement (LGE) [20]. Furthermore, CMR can provide a rapid and comprehensive assessment of aortic vascular health through parameters such as compliance, distensibility, stiffness index, and pulse wave velocity [21]. We hypothesise that significant cardiovascular abnormalities are present in asymptomatic regular cocaine users, and that these will be detectable using CMR.

## Methods

### Ethics Statement

The study was approved by the authors' local institutional ethics committee (Northern Sydney Local Health District Human Research Ethics Committee). Informed written consent was obtained from all study participants. All clinical investigation was conducted according to the principles expressed in the Declaration of Helsinki.

### Sample Population

Twenty subjects with self-reported regular cocaine use (‘users’), and 20 control subjects (‘non-users’) were enrolled. The inclusion criteria were regular frequent cocaine use (defined as at least monthly during the last year) for users and no prior cocaine use for non-users. Exclusion criteria for both groups were known coronary disease or previous MI (self-reported), a contraindication to CMR, or self-reported cocaine use in the 48 hours prior to image acquisition. All participants were over 18 years of age and employed. Recruitment was by ‘word-of-mouth’, and predominantly depended on professional and social networks in an affluent region of Sydney, Australia. Specifically, in contrast to previous studies, we avoided recruitment through drug rehabilitation centres, minimising the number of individuals with a clinical psychiatric diagnosis of cocaine addiction or dependence. All participants completed a questionnaire designed by investigators (SK, SD) at the National Drug and Alcohol Research Centre detailing their demographics, substance use history (including tobacco and alcohol), and cardiac risk factors. Subjects remained anonymous, and personal, demographic, recruitment, and CMR data were all kept strictly confidential.

### Cardiovascular magnetic resonance imaging

All magnetic resonance (MR) data was acquired at a single site using a 1.5 Tesla MR system (GE Healthcare, Milwaukee,WI). Cardiac chamber volumes and myocardial mass were quantified using a short axis stack of Balanced Steady-State Free Precession (bSSFP) cine images (Echo Time (TE) = 1.6 ms; 20 phases; flip angle = 55°). Images were acquired during end-expiratory breath hold. Left and right ventricular volumes, mass and ejection fraction were obtained from the short axis stack by manually contouring end-diastolic and end-systolic endocardial and epicardial borders from the base to the apex. Parameters were indexed to body surface area (BSA) using height and weight obtained from patient questionnaires. Measurements reflecting diastolic function were left ventricular peak filling rate (PFR), time to peak filling rate (TPFR) [22] and left atrial (LA) diameter (measured in the 3-chamber view at end-systole) [23]. Cine image analysis was performed using ReportCard (GE Healthcare, Milwaukee,WI).

**Figure 1:**
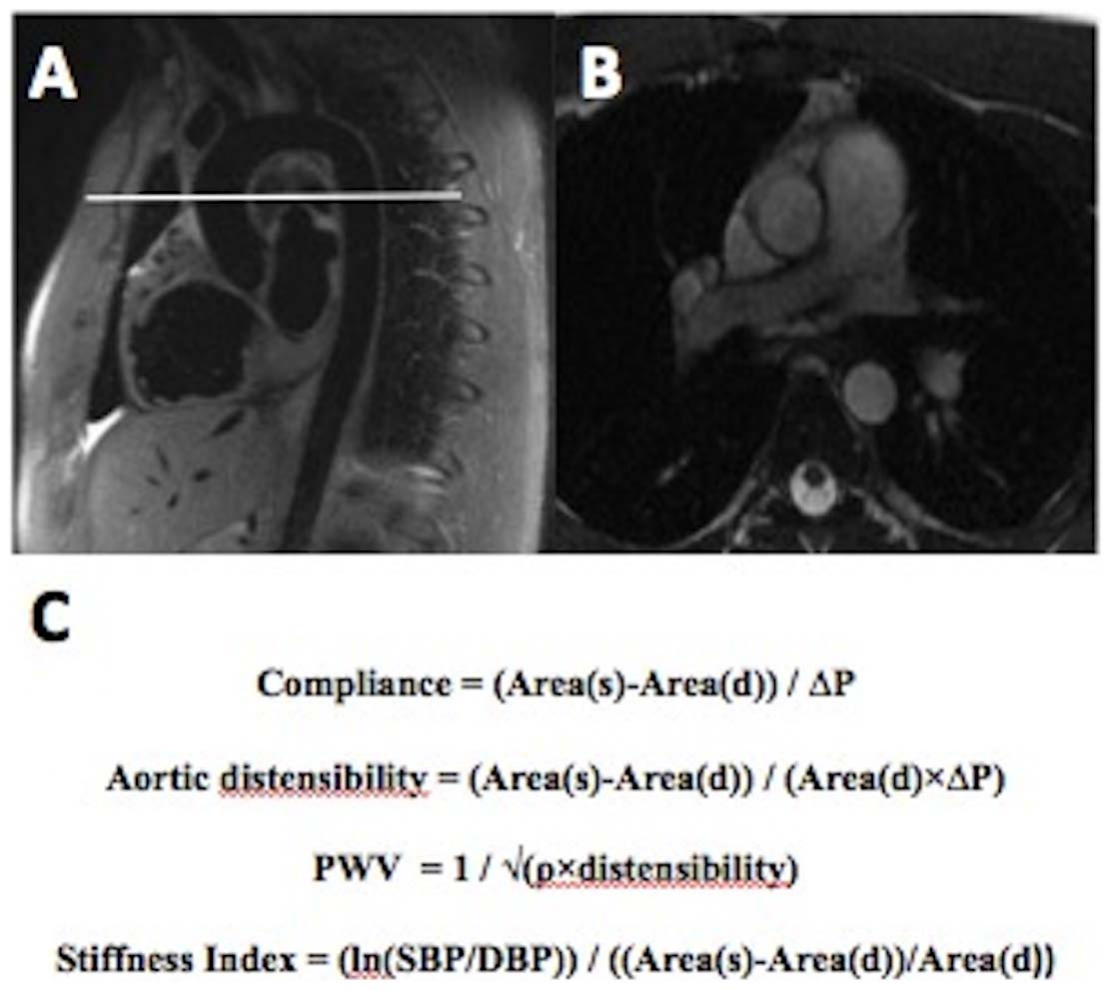
A–B. Scout cardiovascular magnetic resonance image of thoracic aorta demonstrating the planning of a transverse section through proximal descending aorta at the level of the right pulmonary artery. **C.** Arterial stiffness equations. Area(s) = systolic area, area(d) = diastolic area, ΔP = SBP-DBP, ρ = blood density (1059 kg.m^−3^).

Aortic cine images were acquired in the transverse plane, with location of the slice based on scout images (Figure 1A–B) at the level of the right pulmonary artery/pulmonary arch in the proximal descending aorta (PDA). Indices of aortic function were assessed using a prospectively gated cine sequence with a temporal resolution of 30-frames/heartbeat. Sequence parameters were: Field of View (FOV) = 350 mm, TE = 1.4 ms, matrix = 200×200, slice thickness = 8 mm. Gradient echo flow-encoded cine images were acquired with 30 phases and a slice thickness of 4 mm. Aortic cross-sections were manually contoured using Osirix (Rosset, 2004) to calculate the maximal (systolic) and minimal (diastolic) vessel cross-sectional areas. Using these measurements, aortic indices of vascular function at the level of the PDA were calculated using the formulas in Figure 1C [24], [25]. Resting brachial arterial blood pressure was recorded once, by an experienced medical doctor, immediately prior to image acquisition using a manual sphygmomanometer (cuff size 25–34 cm), with the participant in the prone position with arm extended. Measurements of lumen diameter and area, wall thickness, and normalised wall index (NWI = (total vessel area-lumen area)/total vessel area)) [26] were manually contoured using Osirix from high-resolution black blood images taken at the level of the PDA. An average of 3 repeated contour measurements was taken.

LGE imaging was performed 7–15 min after contrast administration (0.2 mmol/kg body weight of gadobenate dimeglumine (Multihance, Bracco Diagnostics, USA). The inversion time was individually adapted to suppress the remote myocardium signal (typical range from 180 to 210 ms). Sequence parameters were: FOV = 350 to 400 mm, Repetition time (TR)/TE = 4.6/1.3 ms, flip angle = 20°, matrix = 256×192, slice thickness = 8 mm. The presence of LGE was assessed by two independent expert observers.

### Statistical Analysis

Statistical analysis was carried out using SPSS 19 (IBM, 2011). All continuous variables are expressed as mean±standard deviation. Categorical variables are expressed as frequencies or as percentages. Normality was checked using the Shapiro-Wilk test. Groups were compared using independent-samples t-test for normally distributed continuous variables, the Mann-Whitney U test for non-normally distributed variables, and using Chi-squared test for binomial or ordinal variables. Our sample size of 20 cocaine users gives us an 88% power to detect a 10% increase in LV mass index with an α-value of 5%. Univariate analyses were initially performed in order to identify which factors were associated with the chosen dependent variables: SBP (systolic blood pressure), aortic stiffness indices and aortic dimensions, LVM (left ventricular mass), LVEDV (left ventricular end-diastolic volume), RVEDV (right ventricular end diastolic volume), LA diameter, PFR, TPFR. Variables entered into the model included cocaine use status (user or non-user), age, gender, BSA, smoking pack years (number of years of smoking equivalent to 1 pack ( = 20 cigarettes) per day) and alcohol (number of standard drinks per week – calculated from self-reported frequency of alcohol consumption and number of standard drinks per session). Backwards step-wise linear regression analyses were then performed in order to identify the most important independent associations of the dependent variables. The unstandardized coefficient B and its 95% confidence interval were recorded. A p-value of <0.05 was considered statistically significant.

## Results

### Participant Characteristics

Table 1 summarises the demographics of both groups. All participants completed the questionnaire and CMR imaging protocol. Both groups were similar in age, gender distribution and self-reported history of dyslipidaemia and diabetes mellitus. No subjects reported taking regular cholesterol modifying drugs, diabetic or anti-hypertensive medications. Cocaine users compared to controls were observed to have a 7% higher BSA (p = 0.03), were more likely to be current smokers (45% vs 5%, p = 0.003), and had higher life-long smoking consumption, although the reported amount was low in both groups (smoking pack years 2.36±5.20 vs 0.01±0.06, p = 0.05). Cocaine users had a similar pattern of frequency of alcohol consumption compared to controls. However, they were more likely to consume a greater number of standard drinks per session. Cocaine users also had a higher self-reported usage of other illicit drugs in their lifetime (95% vs 35%, p<0.001). Concomitant consumption of cocaine, smoking, alcohol and other illicit drugs was not reported.

**Table 1:** Participant characteristics.

	Non Users (n = 20)	Users (n = 20)	p value
Age (yrs)	33±7	37±7	0.07
Male gender	19 (95%)	17 (85%)	0.3
Body surface area (m2)	2.09±0.2	1.95±0.2	0.03
Diabetes	0	0	-
Dyslipidaemia history	0 (0%)	3 (15%)	0.07
Current smoker	1 (5%)	9 (45%)	0.003
Smoking pack years	0.01±0.06	2.36±5.20	0.05
Frequency of alcohol consumption			0.6
Never	2 (10%)	1 (5%)	
Monthly or less	2 (10%)	1 (5%)	
2–4 times per month	5 (25%)	4 (20%)	
2–3 times per week	9 (45%)	8 (40%)	
4+ times per week	2 (10%)	6 (30%)	
Alcoholic drinks per session			0.03
1 or 2	9 (45%)	1 (5%)	
3 or 4	7 (35%)	7 (35%)	
5 or 6	4 (20%)	3 (15%)	
7 or 9	0 (0%)	6 (30%)	
10+	0 (0%)	3 (15%)	
Lifetime use of other drugs	7 (35%)	19 (95%)	<0.001

The median age of first cocaine use was 21 years (range 17–50 yrs.), with the majority of subjects (70%) having used cocaine for over 10 years. Sixty five per cent of cocaine users had a regular pattern of use with a frequency of at least 1–2 days per week. The remaining subjects had a more variable pattern of use, estimated at least monthly over the preceding year. The route of administration most commonly used was nasal insufflation (n = 16, 80%). The other routes were smoking (n = 3, 15%) and injection (n = 1, 5%). All participants had abstained from cocaine use for at least 48 hours prior to imaging (self-reported), satisfying the inclusion criteria, with the average time period between last cocaine use and imaging being 33 days in the user group.

### Haemodynamic and vascular parameters

The haemodynamic and vascular parameters of participants are summarised in Table 2. The results of the multivariate analyses are presented in Table 3 and Table S1 in File S1. Cocaine users had a significantly higher SBP (difference of mean 8 mmHg; p = 0.04), and cocaine use was the most significant predictor of SBP entered into the regression model (B = 7.6 mmHg (95% CI 0.5–14.7), p = 0.036). The relationship between SBP and cocaine use was independent of other covariates including age, gender, BSA, smoking and alcohol consumption. There was a positive relationship between SBP and frequency of cocaine use (B = 0.38 mmHg/times used per week, 95%CI 0.3–7.2, p = 0.04); and a trend towards a significant relationship between SBP and duration of cocaine use (B = 0.5 mmHg/year, 95%CI −0.2–1.0, p = 0.06). No significant difference existed between the user group and controls for diastolic blood pressure (DBP) or heart rate.

**Table 2:** Haemodynamic and vascular parameters.

	Non users	Users	p value
Systolic BP (mmHg)	126±11	134±11	0.04
Diastolic BP (mmHg)	83±13	85±9	0.63
Heart rate (beats/min)	68±9	66±10	0.47
Compliance (cm2.10-2 mmHg-1)*	1.7±0.5	1.3±0.2	0.03
Distensibility (10-3.mmHg-1)*	5.1±1.4	3.8±0.9	0.002
Stiffness Index	2.1±0.6	2.6±0.6	0.005
Pulse wave velocity (m.s-1)	4.4±0.6	5.1±0.6	0.001
Wall thickness (mm)*	2.3±0.3	2.4±0.3	0.8
Normalised Wall Index*	0.34±0.38	0.33±0.03	0.4

* indicates variables analysed by Mann-Whitney U test.

**Table 3:** Statistical analyses of systolic blood pressure and vascular parameters.

Dependent Variables	Variables in Model	Univariate B (95% CI)	p value	Multivariate B (95% CI)	p value
SBP	Cocaine use	7.6 mmHg (0.5, 14.7)	0.036	7.6 mmHg (0.5, 14.7)	0.036
	Age	-	0.13	-	
	Gender	-	0.79	-	
	BSA	-	0.25	-	
	Smoking	-	0.5	-	
	Alcohol	-	0.27	-	
Compliance	Cocaine use	−0.37 cm2.10-2.mmHg-1 (−0.61,−0.13)	0.004	−0.37 cm2.10-2.nnHg-1 (−0.61,−0.13)	0.004
	Age	-	0.08	-	
	Gender	-	0.85	-	
	BSA	-	0.15	-	
	Smoking	-	0.09	-	
	Alcohol	-	0.25	-	
Normalised Wall Index	Cocaine use	-	0.38	-	
	Age	0.001 (−0.003, 0.0)	0.05	−0.001 (−0.003, 0.0)	0.05
	Gender	-	0.6	-	
	BSA	−0.05 (−0.11, 0.001)	0.05	−0.05 (−0.10, 0.001)	0.06
	Smoking	-	0.68	-	
	Alcohol	-	0.3	-	

Cocaine users had a reduction in mean compliance and distensibility in the PDA of 24% and 34% respectively with reciprocal increases in stiffness index and pulse wave velocity compared to non-users (Table 2). These differences remained significant after including the covariates smoking, alcohol, age, gender, and BSA in the model, and regression analyses indicated cocaine use to be an independent predictor of distensibility and compliance. The distribution of compliance measures in the two groups is shown in a scatterplot in Figure S1 in File S1. There was a positive relationship between compliance and both duration and frequency of cocaine use (B = −0.02 cm^2^×10^−2^.mmHg^−1^/year, 95%CI −0.04–−0.01, p = 0.01; B = −0.16 cm^2^×10^−2^.mmHg^−1^/times used per week, 95%CI −0.28–−0.04, p = 0.01, respectively). Of note, the change in aortic stiffness observed in cocaine users occurred without a concomitant increase in vessel wall thickness (Table 2).

### Ventricular structure, mass and function

CMR measures of cardiac mass and function parameters are summarised in Table 4 and Table S2 in File S1. Cocaine users had an 18% greater LVM (p = 0.01), a difference that remained significant after indexing for BSA (p = 0.04). The distribution of indexed LVM in the two groups is shown as a scatterplot in Figure S2 in File S1. All increased LVMs were observed to be in a concentric pattern. The association between cocaine use and indexed LVM was independent of age, gender, smoking and alcohol use. Regression analysis showed cocaine use as the strongest predictor of indexed LVM (B = 7.71 g.m^−2^ (95% CI 0.5–15.0), p = 0.038) (Table 5). Indexed LVM was positively associated with frequency of cocaine use (B = 4.2 g.m^−2^, 95%CI 0.6–7.7, p = 0.02). There was a trend towards a positive association between indexed LVM and duration of cocaine use (B = 0.4 g.m^−2^, 95%CI −0.1–0.9, p = 0.09). All other measures of ventricular volume and function were not significantly different between the groups.

**Table 4:** Cardiac mass, structure and function parameters.

	Non users	Users	p value
LVEDV (ml)	170±24	179±32	0.35
LVEDV index (ml/m2)	88±12	86±5	0.7
LVESV (ml)	65±12	68±15	0.44
LVESV index (ml/m2)	33±6	32±8	0.8
LV Mass (g)	105±16	124±25	0.01
LV Mass index (g/m2)	54±8	61±14	0.04
LVEF (%)	62±4	62±4	0.94
LA size (cm)	3.5±0.3	3.8±0.6	0.04
LA size index (cm/m2)	1.8±0.2	1.8±0.2	0.76
PFR (ml/msec)	0.61±0.17	0.57±0.13	0.38
TPFR (msec)*	248.1±76.3	307.9±207	0.45

* indicates variable analysed by Mann-Whitney U test.

**Table 5:** Statistical analyses of cardiac mass.

Dependent Variables	Variables in Model	Univariate B (95% CI)	p value	Multivariate B (95% CI)	p value
LVM	Cocaine use	18.9 g (5.5, 32.4)	0.007	21.8 g (9.3,34.4)	0.001
	Age	-	0.4	-	
	Gender	−29.0 g (−51.8, −6.2)	0.01	-	
	BSA	41.9 g (7.2, 76.6)	0.02	-	
	Smoking	-	0.34	-	
	Alcohol	-	0.2	-	
LVM index	Cocaine use	7.7 g.m-2 (0.5, 15.0)	0.04	7.71 g.m-2 (0.5,15.0)	0.038
	Age	-	0.47	-	
	Gender	-	0.16	-	
	Smoking	-	0.38	-	
	Alcohol	0.34 g.m-2 (−0.02,0.69)	0.06	-	

Considering the significant findings of elevated SBP and increased LVM in the cocaine user group, we further examined CMR indicators of diastolic function (Table S2 in File S1). Increasing age, but not cocaine use, was a significant predictor of PFR and TPFR. LA diameter was significantly greater in cocaine users compared to non-users (3.8±0.6 cm vs. 3.5±0.3 cm, p = 0.04), however the difference did not remain significant after indexing for BSA, and cocaine use was not a significant contributor to LA diameter in regression analyses.

### Myocardial Assessment

There was no evidence of silent MI as determined by myocardial LGE in any of the study participants. There were also no segmental wall motion abnormalities in either group.

### Recruitment and analysis of matched control group

As this was the first study to attempt to examine the impact of cocaine use in a non-institutionalized user group, the control group was initially randomly recruited from the same community group as the user group. As shown in Table 1, this resulted in suboptimal matching of key covariates in the two groups. We subsequently improved the matching by excluding 4 control individuals who had no smoking or alcohol history, and recruiting an additional 4 individuals who were known smokers, with moderate alcohol consumption. As shown in Table S4 in File S1, the matching of baseline characteristics was much improved by this approach, with no significant differences in smoking, BSA, age, or frequency of alcohol consumption. The key outcome measures in the matched cohort are presented in Tables S5–6 in File S1. SBP, indexed LVM and vascular stiffness (represented by compliance in the PDA) remain significantly different between the user and non-user groups, and cocaine remains an independent predictor of these measures in regression analyses.

## Discussion

This study demonstrates that significant cocaine-related cardiovascular abnormalities are present in otherwise healthy cocaine users who were recruited by word-of-mouth in a well-off area of Sydney, Australia. The key cocaine-associated changes were increased arterial stiffness, elevated SBP and greater LVM in the cocaine user group, findings that were independent of other covariates, including smoking and alcohol consumption. We observed no evidence of silent MI, nor left ventricular diastolic or systolic dysfunction.

In contrast to other previous studies, and in accordance with our aim to recruit healthy individuals who considered themselves ‘social’ users, we did not recruit our cocaine using subjects from drug rehabilitation centres, nor did we require a clinical psychiatric diagnosis of cocaine addiction for enrolment. Instead we relied on ‘word-of-mouth’, which filtered along both social and professional networks to enrol individuals who self-reported their cocaine use. This resulted in our study population reflecting the majority of cocaine users in Australia – young, and employed people who generally snort cocaine on a recreational basis and are considered socially and economically integrated – hence are very relevant to the public health problems associated with cocaine use in our community. This sub-group of users have previously been observed to start using cocaine on a “social-recreational” basis; and do “not expect to become dependent on a drug that is believed to be relatively harmless”, and “have an image of themselves as invincible” [27]. Significantly, it was from a similar group that we witnessed a spate of individuals presenting with large MIs [9]. One of the difficulties with recruiting via the community and not via formal drug rehabilitation centres is the reluctance of those who do not identify as having a formal clinical addiction to volunteer for medical research. The resulting limited number of participants, a potential weakness of the study, was partially counteracted by the power provided by the precision of CMR measurements allowing a detailed and precise anatomical and functional cardiovascular assessment to be performed in a single de-identified visit. For example in our study, the precision of LV mass index measurement with CMR gives us an 88% power to detect a 10% increase in this parameter.

Potential confounding effects related to the higher incidence of smoking, alcohol and illicit drug use in the cocaine user group need to be considered, particularly given the known association of smoking with vascular stiffness and of alcohol with SBP. In our study neither smoking nor alcohol had significant effects on SBP or LVM, although smoking was a predictor of vascular stiffness. In all cases, the relationship of cocaine use with SBP, LVM and vascular stiffness remained significant after including smoking and alcohol consumption in the analyses. We also performed secondary analyses examining the impact of cocaine use on SBP, LVM and arterial compliance after excluding smokers (Table S3 in File S1). Aortic compliance remained significantly less in cocaine users versus controls. The remaining parameters showed similar trends to the primary analyses, but statistical power was limited due to reduced numbers. In a separate secondary analysis we excluded heavier drinkers (those who drank >10 standard drinks per week) (Table S3 in File S1). SBP, LVM, and arterial compliance differences trended in the same direction as the primary analyses, but were not significant. Finally, we performed additional analyses on a strategically matched control group, which further supported initial data, supporting cocaine use as an important predictor of SBP, LVM and vascular stiffness (Tables S4–6 in File S1).

The increase in SBP in cocaine users was present despite a 48-hour period of abstinence, and was independent of age, BSA, gender, alcohol and smoking history. Although cocaine ingestion causes acute rapid and extreme increases in blood pressure via mechanisms including potent sympathomimetic properties and arterial vasoconstriction, the effects over the longer term are not well understood. One possible mechanism for the persistence of systolic hypertension in cocaine users observed in this study is an effect of cocaine on arterial stiffness - a hypothesis that is supported by our results. Cocaine users in our cohort had significant decreases in compliance and distensibility, with reciprocal increases in stiffness index and pulse wave velocity compared to controls. This is similar to a report of altered aortic properties in long-term crack cocaine abusers demonstrated by echocardiography [28]. As in this study, our aortic stiffness parameters correlated with duration and frequency of cocaine use. Such an association is consistent with a cumulative effect of cocaine exposure on the vessel wall. Interestingly, the lack of increased wall thickness in our cocaine users suggests that the arterial stiffness effect of cocaine is via alteration of the proportion of elastic versus non-elastic content ratio, rather than vascular smooth muscle hypertrophy. It should also be noted that arterial stiffness might be overestimated in this study by using brachial pulse pressure measurements instead of central [29].

A major finding of this study is that the cocaine users displayed significantly greater LVMs than control subjects, and that cocaine use was the most significant contributor to LVM in the statistical model. The observation of increased LVM in cocaine users supports and extends previous reports of this association in cohorts with heavier reported use of cocaine [13], [14]. The correlation of duration and frequency of cocaine use with LVM also supports this relationship. It should be noted, however, that all individual LVM values of both groups fell into the normal range of values according to age and gender [30]. To ensure we did not underestimate LVM, we additionally indexed LVM to height to the power of 2.7. This indexation method has been shown to offer a more accurate estimation of LVM in overweight and obese subjects [31]. However, using LVM indexed in this fashion did not alter our findings thus has not been presented.

Despite the increased SBP, LVM and aortic stiffness, we did not detect any differences in diastolic function in cocaine users. Non-invasive quantitative assessment of diastolic function is mostly performed with transthoracic echocardiography in clinical practice, however CMR has previously been validated and used to evaluate LV relaxation and stiffness abnormalities using a combination of left atrial size measurement, phase-contrast evaluation of transmitral flow and LV filling (PFR and TPF) [22]. We did observe an association of decreasing diastolic function with age, supporting the use of the measured CMR parameters, however it is possible that CMR does not provide the sensitivity of echocardiography. Our study may also be underpowered to detect these diastolic parameters. Indeed, cocaine use has previously been associated with diastolic dysfunction measured by echocardiography, even in the absence of left ventricular hypertrophy [14].

The lack of LGE evidence of silent MI or systolic dysfunction in this study contrasts with previous reports in cocaine addicts [11], [12], [32]. Indeed, in a recent study by Aquaro et al. [11], 47% of subjects with a clinical diagnosis of cocaine addiction had myocardial fibrosis demonstrated by LGE, with 32% of these being in an ischaemic pattern. Differences in cohort characteristics may explain at least part of this discrepancy, with Aquaro et al. concluding that the results “may not be applicable to most recreational (‘weekend’) cocaine users”. Indeed, there was a much higher reporting of intravenous administration of cocaine (33% versus 5%) in their cohort; and the rate of clinically diagnosed polydrug addiction was high at 53%. This latter figure is difficult to compare to our study, which only identified ‘use’ of other illicit substances, not ‘addiction’. Alcohol co-consumption appears to be an important factor in the toxic effects of cocaine, potentially by altered hepatic biotransformation of cocaine and the production of the toxic metabolite cocaethylene, as well as inhibition of cocaine clearance [33], [34]. Interestingly, the lack of LGE present in our cohort appears to contradict this proposed association given our subjects reported drinking an average of 18 drinks per week.

The main limitation of this study - its sample size - has already been discussed. In addition, as the subjects were completely anonymous, and had only one study visit, we relied on self-reporting in the study questionnaire for information on baseline characteristics. The decision to use this approach was based on previous work by members of our team, which found that self-reported data on drug use patterns are sufficiently reliable and valid [35], [36]. Data on previous blood pressure measurements of participants, particularly prior to the commencement of cocaine use, or equivalent time period in the control group, would also assist in interpreting the data. Designing a questionnaire to identify the relative frequency of concomitant use of alcohol and cocaine may also have been helpful to understand the mechanism of cardiovascular toxicity given the theoretical pharmacological interaction.

This is the first study to examine the cardiovascular effects of cocaine use in ‘non-addict’ individuals who consider themselves ‘social’ users (with other studies focusing on addicts, recruited through treatment centres). Although this group is thought to represent a large majority of cocaine users in our community, the illicit nature of cocaine use makes recruitment notoriously difficult. CMR provided the power to study this group and compared with the non-user control cohort, cocaine users had increased aortic stiffness, an 8 mmHg higher SBP, and a 19 g greater left ventricular mass. These measures are all well known risk factors for premature cardiovascular events, highlighting the dangers of cocaine use even in a ‘social’ setting.

## Supporting Information

File S1 **This file contains Tables S1–S6 and Figures S1–S2.** Table S1, Statistical analyses of additional vascular parameters. Table S2, Statistical analyses of additional cardiac structure and diastolic function parameters. Table S3, Secondary analyses of non smokers and low consumption drinkers in subjects and controls. Table S4, Baseline characteristics of matched cohort. Table S5, Comparison of key outcome measures in matched cohort. Table S6, Univariate and multivariate regression analyses on key outcome measures in matched cohort. Figure S1, Distribution of compliance values in subjects and controls. Figure S2, Distribution of indexed left ventricular mass values in subjects and controls.
(DOCX)
