# Supplementary material for: Regular Cocaine Use Is Associated with Increased Systolic Blood Pressure, Aortic Stiffness and Left Ventricular Mass in Young Otherwise Healthy Individuals
Source: PLoS One. 2014 Apr 9;9(4):e89710. doi: 10.1371/journal.pone.0089710 (PMC3981670; doi:10.1371/journal.pone.0089710)
Supplement: File S1 — This file contains Tables S1–S6 and Figures S1–S2. Table S1, Statistical analyses of additional vascular parameters. Table S2, Statistical analyses of additional cardiac structure and diastolic function parameters. Table S3, Secondary analyses of non smokers and low consumption drinkers in subjects and controls. Table S4, Baseline characteristics of matched cohort. Table S5, Comparison of key outcome measures in matched cohort. Table S6, Univariate and multivariate regression analyses on key outcome measures in matched cohort. Figure S1, Distribution of compliance values in subjects and controls. Figure S2, Distribution of indexed left ventricular mass values in subjects and controls. (DOCX) [file pone.0089710.s001.docx]

**Table S1.** Statistical analyses of additional vascular parameters

| **Dependent Variables** | **Variables in Model** | **Univariate B (95% CI)** | **p value** | **Multivariate B (95% CI)** | **p value** |
| --- | --- | --- | --- | --- | --- |
|  |  |  |  |  |  |
| Distensibility | Cocaine use | -1.32mmHg-1 (-2.07,-0.57) | 0.001 | -1.18mmHg-1 (-0.49) | 0.001 |
|  | Age | -9.2mmHg-1 (-9.2,-0.001) | 0.001 | -0.07mmHg-1 (-0.11,-0.02) | 0.01 |
|  | Gender | - | 0.12 | -1.34mmHg-1 (-2.43,-0.25) | 0.02 |
|  | BSA | -2.79mmHg-1 (-4.77,-0.80) | 0.01 | - |  |
|  | Smoking | - | 0.13 | - |  |
|  | Alcohol | - | 0.06 | - |  |
|  |  |  |  |  |  |
| Stiffness index | Cocaine use | 0.55 (0.17, 0.92) | 0.005 | - |  |
|  | Age | 0.045 (0.02, 0.07) | < 0.001 | 0.041(0.02,0.062) | < 0.001 |
|  | Gender | - | 0.19 | - |  |
|  | BSA | 1.28 (0.33, 2.23) | 0.01 | 1.28(0.49,2.07) | 0.002 |
|  | Smoking | - | 0.16 | - |  |
|  | Alcohol | - | 0.17 | - |  |
|  |  |  |  |  |  |
| Pulse wave velocity | Cocaine use | 2.24m.s-1 (0.97, 3.51) | 0.001 | - |  |
|  | Age | 0.18m.s-1 (0.10, 0.26) | < 0.001 | 0.16m.s-1 (0.09, 0.23) | < 0.001 |
|  | Gender | - | 0.18 | - |  |
|  | BSA | 4.77m.s-1 (1.42, 8.12) | 0.006 | 4.76m.s-1 (2.2,7.32) | 0.001 |
|  | Smoking | - | 0.12 | - |  |
|  | Alcohol | - | 0.08 | 0.002 (0,0.003) | 0.03 |
|  |  |  |  |  |  |
| Wall Thickness | Cocaine use | - | 0.8 | - |  |
|  | Age | - | 0.4 | - |  |
|  | Gender | - | 0.3 | - |  |
|  | BSA | - | 0.98 | - |  |
|  | Smoking | - | 0.19 | - |  |
|  | Alcohol | - | 0.4 | - |  |

**Table S2.** Statistical analyses of additional cardiac structure and diastolic function parameters

| **Dependent Variables** | **Variables in Model** | **Univariate B (95% CI)** | **p value** | **Multivariate B (95% CI)** | **p value** |
| --- | --- | --- | --- | --- | --- |
|  |  |  |  |  |  |
| LVEDV | Cocaine use |  | 0.35 | - |  |
|  | Age |  | 0.33 | - |  |
|  | Gender | -35.7ml (-64.2, -7.3) | 0.015 | 28.6ml (-0.7,58.0) | 0.055 |
|  | BSA | 44.0ml (-0.3, 88.2) | 0.051 | - |  |
|  | Smoking |  | 0.4 | - |  |
|  | Alcohol |  | 0.4 | - |  |
|  |  |  |  |  |  |
| LVEDV index | Cocaine use |  | 0.7 | - |  |
|  | Age |  | 0.3 | - |  |
|  | Gender |  | 0.35 | - |  |
|  | Smoking |  | 0.63 | - |  |
|  | Alcohol |  | 0.4 | - |  |
|  |  |  |  |  |  |
| LA diameter | Cocaine use | 0.31cm (0.01, 0.61) | 0.041 | - |  |
|  | Age | 0.03cm (0.01, 0.05) | 0.006 | 0.03cm (0.01, 0.04) | 0.003 |
|  | Gender |  | 0.11 | - |  |
|  | BSA | 1.15cm (0.45, 1.84) | 0.002 | 1.1cm (0.47, 1.7) | 0.001 |
|  | Smoking |  | 0.4 | - |  |
|  | Alcohol |  | 0.4 | - |  |
|  |  |  |  |  |  |
| LA diameter index | Cocaine use |  | 0.76 | - |  |
|  | Age | 0.01cm.m-2 (0.003, 0.02) | 0.01 | 0.01cm.m-2 (0.002, 0.02) | 0.014 |
|  | Gender |  | 0.87 | - |  |
|  | Smoking |  | 0.12 | - |  |
|  | Alcohol |  | 0.5 |  |  |
|  |  |  |  |  |  |
| PFR | Cocaine use |  | 0.38 | - |  |
|  | Age | -0.1ml.msec-1 (-002, -0.03) | 0.005 | -0.08ml.msec-1 (-0.14,-0.02) | 0.008 |
|  | Gender |  | 0.89 | - |  |
|  | BSA |  | 0.29 | - |  |
|  | Smoking |  | 0.6 | - |  |
|  | Alcohol |  | 0.08 | - |  |
|  |  |  |  |  |  |
| TPFR | Cocaine use |  | 0.24 | - |  |
|  | Age | 109.41msec (52.48, 166.34) | < 0.001 | 109.0msec (52.0,166.0) | < 0.001 |
|  | Gender |  | 0.55 | - |  |
|  | BSA |  | 0.31 | - |  |
|  | Smoking |  | 0.37 | - |  |
|  | Alcohol |  | 0.5 | - |  |

**Table S3:** Secondary analyses of non smokers and low

consumption drinkers in subjects and controls

| **Non Smokers** | **Subjects (n=11)** | **Controls (n=19)** | **p value** |
| --- | --- | --- | --- |
| Systolic BP | 135±13 | 128±11 | 0.1 |
| LVM Indexed | 56±8 | 54±8 | 0.4 |
| Aortic Compliance | 3.9±1.1 | 5.1±1.1 | 0.03 |
|  |  |  |  |
|  |  |  |  |
| **Low Consumption Drinkers** | **Subjects (n=6)** | **Controls (n=14)** | **p value** |
| Systolic BP | 136±7 | 128±10 | 0.08 |
| LVM Indexed | 60±16 | 54±7 | 0.26 |
| Aortic Compliance | 3.9±1.0 | 4.8±1.4 | 0.2 |

**Table S4:** Baseline characteristics of matched cohort

|  | **Non Users (n=20)** | **Users (n=20)** | **p value** |
| --- | --- | --- | --- |
|  |  |  |  |
| Age (yrs) | 34 ± 7 | 37 ± 7 | 0.1 |
| Male gender | 19 (95%) | 17 (85%) | 0.3 |
| Body mass index | 25.6 ± 2.6 | 27.1 ± 3.5 | 0.1 |
| Diabetes | 0 | 0 | - |
| Dyslipidaemia history | 1 (5%) | 3 (15%) | 0.3 |
| Current smoker | 5 (25%) | 9 (45%) | 0.19 |
| Smoking pack years | 2.2 ± 5.2 | 2.4 ± 5.1 | 0.9 |
| Frequency of alcohol consumption |  |  | 0.6 |
| Never | 2 (10%) | 1 (5%) |  |
| Monthly or less | 2 (10%) | 1 (5%) |  |
| 2-4 times per month | 4 (20%) | 4 (20%) |  |
| 2-3 times per week | 10 (50%) | 8 (40%) |  |
| 4+ times per week | 2 (10%) | 6 (30%) |  |
| Alcoholic drinks per session |  |  | 0.03 |
| 1 or 2 | 8 (40%) | 1 (5%) |  |
| 3 or 4 | 4 (20%) | 7 (35%) |  |
| 5 or 6 | 5 (25%) | 3 (15%) |  |
| 7 or 9 | 1 (5%) | 6 (30%) |  |
| 10+ | 2 (10%) | 3 (15%) |  |
| Lifetime use of other drugs | 8 (40%) | 19 (95%) | < 0.001 |

**Table S5**: Comparison of key outcome measures in matched cohort

|  | **Non users** | **Users** | **p value** |
| --- | --- | --- | --- |
|  |  |  |  |
| Systolic BP (mmHg) | 124 ± 11 | 134 ± 11 | 0.006 |
| Compliance (cm2.10-2mmHg-1)* | 1.9 ± 0.6 | 1.3 ± 0.2 | 0.004 |
| LV Mass index (g/m2) | 53.0 ± 8.9 | 61.4 ± 14.0 | 0.03 |

* indicates variable analysed by Mann-Whitney U test

**Table S6**: Univariate and multivariate regression analyses on key outcome measures in matched cohort

| **Dependent Variables** | **Variables in Model** | **Univariate B (95% CI)** | **p value** | **Multivariate B (95% CI)** | **p value** |
| --- | --- | --- | --- | --- | --- |
|  |  |  |  |  |  |
| SBP | Cocaine use | 10.0mmHg (2.99, 16.91) | 0.006 | 10.0mmHg (2.99, 16.91) | 0.006 |
|  | Age | - | 0.05 | - |  |
|  | Gender | - | 0.61 | - |  |
|  | BSA | - | 0.28 | - |  |
|  | Smoking | - | 0.79 | - |  |
|  | Alcohol | - | 0.9 | - |  |
|  |  |  |  |  |  |
| Compliance | Cocaine use | -0.54cm2.10-2.mmHg-1 (-0.82,-0.27) | <0.001 | -0.67cm2.10-2.nnHg-1 (-0.94,-0.38) | <0.001 |
|  | Age | - | 0.09 | - |  |
|  | Gender | - | 0.71 | - |  |
|  | BSA | - | 0.27 | - |  |
|  | Smoking | - | 0.93 | - |  |
|  | Alcohol | - | 0.51 | - |  |
|  |  |  |  |  |  |
| LVM index | Cocaine use | 8.4g.m-2 (0.9, 15.9) | 0.03 | 8.4g.m-2 (0.9, 15.9) | 0.03 |
|  | Age | - | 0.49 | - |  |
|  | Gender | - | 0.36 | - |  |
|  | Smoking | - | 0.9 | - |  |
|  | Alcohol | - | 0.14 | - |  |


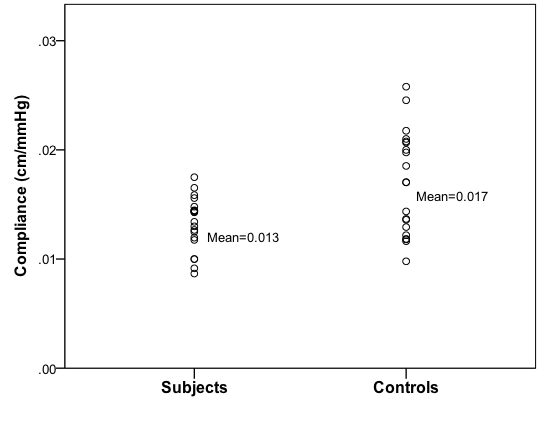


**Figure S1.** Distribution of compliance values in subjects and

controls


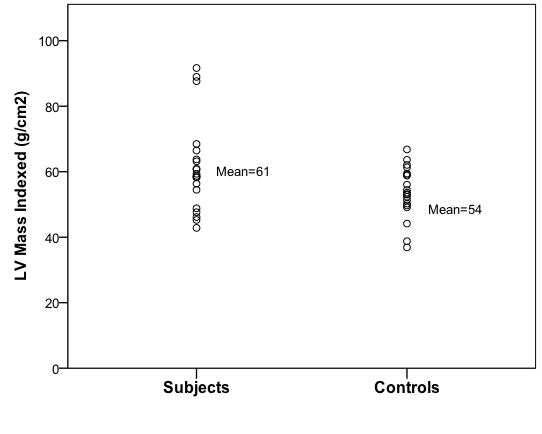


**Figure S2.** Distribution of indexed left ventricular mass

values in subjects and controls
